# Controlling Nanostructure in Inkjet Printed Organic Transistors for Pressure Sensing Applications

**DOI:** 10.3390/nano11051185

**Published:** 2021-04-30

**Authors:** Matthew J. Griffith, Nathan A. Cooling, Daniel C. Elkington, Michael Wasson, Xiaojing Zhou, Warwick J. Belcher, Paul C. Dastoor

**Affiliations:** 1 Centre for Organic Electronics, University of Newcastle, University Drive, Callaghan, NSW 2308, Australia; nathan.cooling@newcastle.edu.au (N.A.C.); daniel.elkington@newcastle.edu.au (D.C.E.); michael.wasson@uon.edu.au (M.W.); xiaojing.zhou@newcastle.edu.au (X.Z.); warwick.belcher@newcastle.edu.au (W.J.B.); paul.dastoor@newcastle.edu.au (P.C.D.); 2 School of Aeronautical, Mechanical and Mechatronic Engineering, University of Sydney, Camperdown, NSW 2006, Australia

**Keywords:** inkjet printing, organic electronics, pressure, sensor, transistor

## Abstract

This work reports the development of a highly sensitive pressure detector prepared by inkjet printing of electroactive organic semiconducting materials. The pressure sensing is achieved by incorporating a quantum tunnelling composite material composed of graphite nanoparticles in a rubber matrix into the multilayer nanostructure of a printed organic thin film transistor. This printed device was able to convert shock wave inputs rapidly and reproducibly into an inherently amplified electronic output signal. Variation of the organic ink material, solvents, and printing speeds were shown to modulate the multilayer nanostructure of the organic semiconducting and dielectric layers, enabling tuneable optimisation of the transistor response. The optimised printed device exhibits rapid switching from a non-conductive to a conductive state upon application of low pressures whilst operating at very low source-drain voltages (0–5 V), a feature that is often required in applications sensitive to stray electromagnetic signals but is not provided by conventional inorganic transistors and switches. The printed sensor also operates without the need for any gate voltage bias, further reducing the electronics required for operation. The printable low-voltage sensing and signalling system offers a route to simple low-cost assemblies for secure detection of stimuli in highly energetic systems including combustible or chemically sensitive materials.

## 1. Introduction

The emergence of functional electronic devices created by low-cost printing techniques has created substantial interest in organic semiconductors as a particularly attractive class of materials for printed electronics [1,2,3,4]. The interest in organic semiconductors arises from two key features of these materials. Firstly, modification of their molecular structure to tune their chemical, physical and electronic properties can be achieved relatively easily through conventional wet chemistry procedures [5,6,7,8]. Secondly, organic materials, in direct contrast to many of their inorganic counterparts, can be dissolved in solutions to create functional inks. This property enables printing of functional electronic devices directly onto mechanically flexible substrates at high speeds across large areas using cheap roll-to-roll (R2R) processing techniques [9,10,11,12]. These two key features have seen the deployment of organic semiconductors across a range of printed electronics applications, including solar cells, [13,14,15,16] transistors, [17,18,19,20] and sensor devices [21,22,23]. The materials have shown particular promise in pressure sensing applications, where most of the focus has been placed on low detection thresholds for tactile applications [24,25,26]. However, secure control of the timing and sequencing of energetic pressure events using electronic detectors is another important consideration in a range of different industries. Therefore, the development of a low-cost pressure sensing element that can rapidly and reliably detect the high-pressure impulses associated with energetic events (such as explosions) is therefore an area that requires further attention.

Conventional electronic detectors meet a key requirement of energetic event detection technology by providing precise timing control on the sub-millisecond time scale and simple multiplexing through the programming of delay times of individual events [27]. However, these detectors are expensive, involve components that operate at high voltages and are prone to failure through inadvertent short-circuits or false triggering from electromagnetic interference [28,29]. Some of these issues have been addressed with modern developments such as wireless initiation signals and RFID safety mechanisms. However, these features add significant complexity and cost to the sensing and signalling apparatus [30]. By contrast, physical transmission fuses are much more robust, immune to electromagnetic interference, and can be manually daisy-chained to initiate serial arrays of events [31]. Upon ignition of transmission fuse, an advancing high energy wavefront propagates down the tube and subsequently triggers a secondary detection element at a defined rate [32]. The high energy impulse consists of several detectable physical stimuli, including light, pressure, heat and gas [33,34,35]. Development of new materials or fabrication techniques that can produce a device to integrate a secure electronic signalling output with the simplicity of the transmission fuse signal input would be of major economic and technical significance.

Organic thin film transistors (OTFTs) are a promising low-cost pathway for creating sensors which can respond to a physical stimuli and produce an electronic output that seamlessly integrates with additional circuitry [36]. Furthermore, OTFTs can be fabricated using low-temperature digital printing processes on a variety of substrates, including low cost and mechanically flexible polymers [37]. Miniaturization to the micrometre scale or smaller has been achieved for these printed devices [1,26] as well as other circuitry components such as diodes and resistors, [38] and thus multicomponent printed circuits prepared entirely from organic inks are readily achievable with this technology [39]. There are several different printing technologies that have been examined for fabricating organic electronic inks. Amongst these options, droplet-based techniques have proven highly popular due to the ability to rapidly prototype by depositing computer-controlled patterns with micrometre resolution onto flexible substrates. Nanostructured functional inks have attracted significant attention for this purpose due to the ability to tune the functionality of materials by altering their morphology. Accordingly, there has been a recent surge in research outputs from nanostructured inks with conducting, semiconducting, magnetic, and piezoelectric properties [40,41,42,43]. Critically, these organic electronic devices are much cheaper than standard inorganic electronics due to the solution-based fabrication, which is a highly desirable feature in a detector unit that will be destroyed upon detonation. Moreover, some types of OTFTs can operate at much lower input voltages than their inorganic counterparts, [17] and thus they are ideally suited for deployment in industrial environments that require low signal voltages to minimize stray electromagnetic signals [5]. OTFT arrays have already shown great promise as signal transducers to convert pulsed light [44,45] and pressure [46,47] stimuli into electronic output signals. Coupled with the low operating power requirements, such devices are ideally suited to convert signal transmission fuse pressure pulses into an electronic output which can then be integrated with timing circuits.

Pressure signals from transmission fuses can be coupled to an electronic component using quantum tunnelling composite (QTC) materials. These materials employ a low-cost flexible matrix into which conducting nanoparticles are impregnated. Upon application of pressure to the composite, the flexible matrix compresses, and high conductivities can be attained through quantum tunnelling of electrons between the conductive components [24,48,49]. Previously, we have presented preliminary results on a prototype OTFT fabricated using spin coating and vacuum-based deposition procedures. This prototype employed a QTC switch in an external circuit as a sensing mechanism [23]. In this paper, we extend those preliminary results and report the full development of an inkjet-printed fully integrated organic electronic pressure sensing and signalling system, fabricated by inserting a printable solution-processed QTC sensing unit as a new layer in the OTFT structure. This sensing unit modulates the gate voltage in response to the pressure output from transmission fuses. We first establish that the QTC material reversibly responds to the pressure wave from the transmission fuse by switching between conducting and insulating states. We then demonstrate the production of a fully printed OTFT with an integrated QTC sensing element. As such, this new sensing and signalling system meets the three critical requirements for an energetic event initiation system: (1) low cost and disposability, (2) low operating power, and (3) an ability to electronically trigger detonation with millisecond timing.

## 2. Materials and Methods

### 2.1. QTC Pressure Sensing Element Calibration

Commercial QTC pressure sensitive samples for benchmarking were purchased from Zoflex (ZL45.1). The material consists of an insulating organosiloxane elastomer blend into which conductive silver nanoparticles were dispersed during casting. QTC samples for solution casting into the integrated OTFT sensor were prepared by incorporating graphite nanopowder into a silicone elastomer matrix (Slygard 184, Dow Corning, Midland, MI, USA) in a ratio of 35% by weight. The elastomer was prepared using a 10:1 ratio of base to curing agent, producing a solution-based QTC with sufficient viscosity for casting and coating procedures. The conductivity response of the QTC materials to applied pressure was measured through the impingement of a known mass onto the QTC material through a piston with a fixed area of 0.4 cm^2^. The applied force was determined from the mass, with the pressure subsequently computed by normalizing the force to the piston area. The resistivity of the QTC material in response to each applied mass was determined with a multimeter using fixed contacts for all weights to eliminate variations in contact resistance. The QTC samples were allowed to return to the decompressed state for 30 s prior to the application of each new mass.

### 2.2. OTFT Construction

Standard reference devices were prepared on glass substrates coated with pre-patterned indium-doped tin oxide (ITO) to produce a source-drain channel 3 mm by 20 µm (Kintec Company, R_sheet_ < 15 Ω m^−2^). The semiconducting polymer layer was deposited by spin-coating a 20 mg mL^−1^ chloroform solution of poly(3-hexylthiophene) (P3HT) (Luminescence Technology Corporation) onto the substrates at 2000 rpm. After drying at 45 °C for 15 min, typical P3HT film thicknesses, measured using an Alpha Step 500 profilometer, were 100 nm. The dielectric layer was subsequently deposited by spin-coating an 80 mg mL^−1^ solution of poly(vinylpyrrolidinone) (PVP) (Sigma-Aldrich, St. Louis, MO, USA) in ethanol onto the P3HT films at 2000 rpm. The outer edges of the electrodes were then exposed by removing the polymer layers with an ethyl acetate rinse, and the PVP layer was dried at 85 °C for 45 min. Typical PVP layers were determined to be 400 nm from profilometry measurements. The gate electrode was subsequently fashioned by drop-casting an aqueous solution of poly(3,4-ethylenedioxythiophene) (PEDOT) doped with poly(styrenesulfonate) (PSS) (HC Starck, Munich, Germany) onto the PVP, followed by drying at 45 °C for 15 min.

Printed OTFT devices were fabricated using a Dimatix DMP2800 inkjet printer (Fuji Xerox, Tokyo, Japan) for each layer. The P3HT ink was prepared with a solids content of 1.3% by weight in a 1:1:1 ternary solvent mixture of indan, o-xylene and tetralin. A PVP interlayer was then deposited from a 40 mg mL^−1^ solution prepared in 2-propanol to enable wetting of the dielectric layer on the P3HT. The PVP dielectric was printed from a 40 mg mL^−1^ solution prepared in hexanol with the addition of 0.7% poly(melamine-co-formaldehyde) added as a cross linking agent, which has been previously reported to chemically crosslink PVP with high efficiency [50]. The PEDOT:PSS gate electrode was then printed using an ink prepared by mixing PH1000 high conductivity PEDOT:PSS ink (Heraeus, Hanau, Germany) with 0.1% ZONYL FSO surfactant (Sigma Aldrich, St. Louis, MO, USA).

### 2.3. OTFT Characterisation

A Keithley 2400 source measure unit controlled by customized LabVIEW software was employed to systematically adjust the voltage between source and drain electrodes and measure the resultant drain current at a fixed gate voltage. A second Keithley 2400 unit (Solon, OH, USA) was used to independently control the voltage between the gate and source electrodes. For initial experiments involving the QTC pressure sensor as a separate switch element, the gate voltage was applied through a voltage divider circuit consisting of a 1 kΩ pull-down resistor connected in series with the QTC pressure sensor as described previously [27]. For experiments where the QTC sensing element was incorporated into the transistor structure, the transmission fuse was terminated into a pneumatically sealed piston which impinged directly onto the laminated QTC element whilst the gate voltage was applied directly to the PEDOT gate electrode.

## 3. Results and Discussion

### 3.1. Characterisation of Transmission Fuse Output and QTC Sensor Response

**Figure 1:**
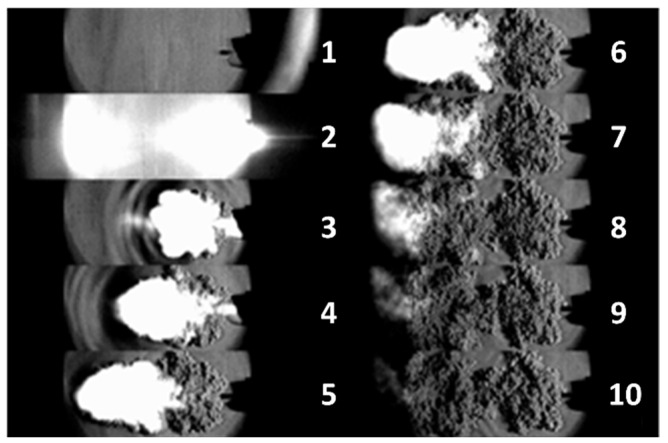
Images of the stimuli emanating from the transmission fuse following initiation recorded with Schlieren optics at a time resolution of 1 µs between images. 1. Tube image prior to initiation. 2. Initial light emission preceding shock front. 3, 4. Pressure wave ripples together with white hot gas emission. 5–10. Gas emission followed by other combustion products.

In order to understand the evolution and propagation of the wavefront down the transmission fuse, a high speed digital camera was used along with typical Schlieren optics [51] to create a time-resolved series of images of the high energy output. The images shown in Figure 1 highlight the series of detectable physical stimuli that comprise the energy output from the transmission fuse. This process commences with an initial light pulse (image 2), followed by a pressure wave (ripples in images 3 and 4), gas output (bright spots in images 3–8) and finally the release of other combustion products (images 5–10). These images are consistent with previous studies of transmission fuse outputs, which reported similar stimuli emanating from the transmission fuse, with a total energy of approximately 1.5 J [35]. The Schlieren images confirm that a strong shock wave is the first physical stimulus released after the initial light pulse, and thus is an ideal candidate for fast detection as part of an initiation system.

**Figure 2:**
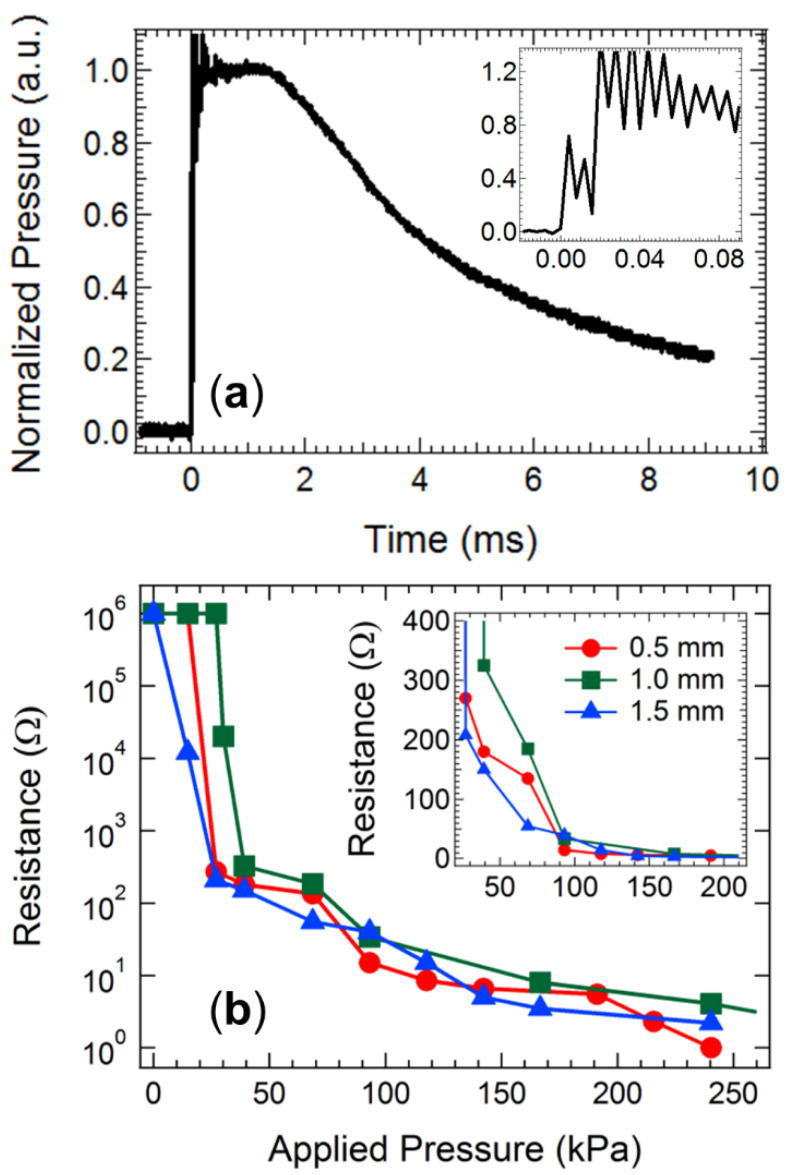
(**a**) The pressure output measured with a transducer and charge amplifier after initiation of the transmission fuse. The inset displays a magnification of the pressure waveform on the time scale up to 80 µs. (**b**) The conductivity response of QTC samples with various thickness to applied pressure. The inset displays a magnified view of the low-pressure region.

The temporal characteristics of the pressure wave released from the transmission fuse were measured using a piezoelectric transducer coupled to a charge amplifier. Figure 2a displays the measured pressure pulse, which exhibits a rise time of ~10 µs to peak pressure. The magnitude of the pressure peak was found to be somewhat variable across repeated trials and measurement geometries but showed good agreement with both the measurements [52] and modelling simulations [53] of other groups, who report peak pressures between 5–20 MPa. This value represents the maximum input pressure available to trigger a subsequent OTFT detector assembly. The data displayed in the inset of Figure 2a also reveal a periodic oscillation in the pressure pulse on the faster time scale (up to 80 µs after initiation), which causes the output pressure to fluctuate from the mean value by ~25% at a frequency of 125 kHz. This phenomenon was also observed in modelling studies of the shock tube signal propagation, implying that it may be a feature inherent to the chemical reaction propagation through the transmission fuse rather than detector noise, although the physical origin of the fluctuation remains unclear [53]. The high frequency fluctuation could potentially influence the pressure level available to trigger a detector assembly. Thus, taking the lower limit of the shock wave pulse and reducing it by 25% to account for the high frequency fluctuations, a conservatively determined maximum value of 4 MPa is available for triggering subsequent detector equipment. Furthermore, the maximum pressure is observed to remain steady for a time of 1.5 ms, after which it steadily decays. Any detector assembly triggering from this pressure input must therefore initiate at input pressures below 4 MPa and within 1.5 ms of receiving the initial pressure pulse.

The conductivity response of the QTC pressure sensing element unit was subsequently examined to ensure it responded appropriately to pressure values below 4 MPa. The conductivity of commercial QTC sensing samples with assorted thicknesses in response to variations in applied pressure is shown in Figure 2b. The conductivity exhibits clear switching behaviour in response to applied pressure, with a threshold value below which the rubber remains in a resistive state (R > 10 kΩ) and above which the rubber transitions to a conductive state (R < 300 Ω). The QTC component switches to a high conductivity state at applied pressure values of 50–100 kPa, easily below the available limit of 4 MPa released from the transmission fuse. This result confirms that the cheap and disposable QTC element is an ideal sensing component for deployment in a low-cost detector unit. We have previously employed this material as an external pressure switch, and the current data will form the baseline for acceptable pressure switching conductivity behaviour for custom-prepared QTC materials that can be processed from solution.

### 3.2. Characterisation of the Printed OTFT Sensing and Signalling System

**Figure 3:**
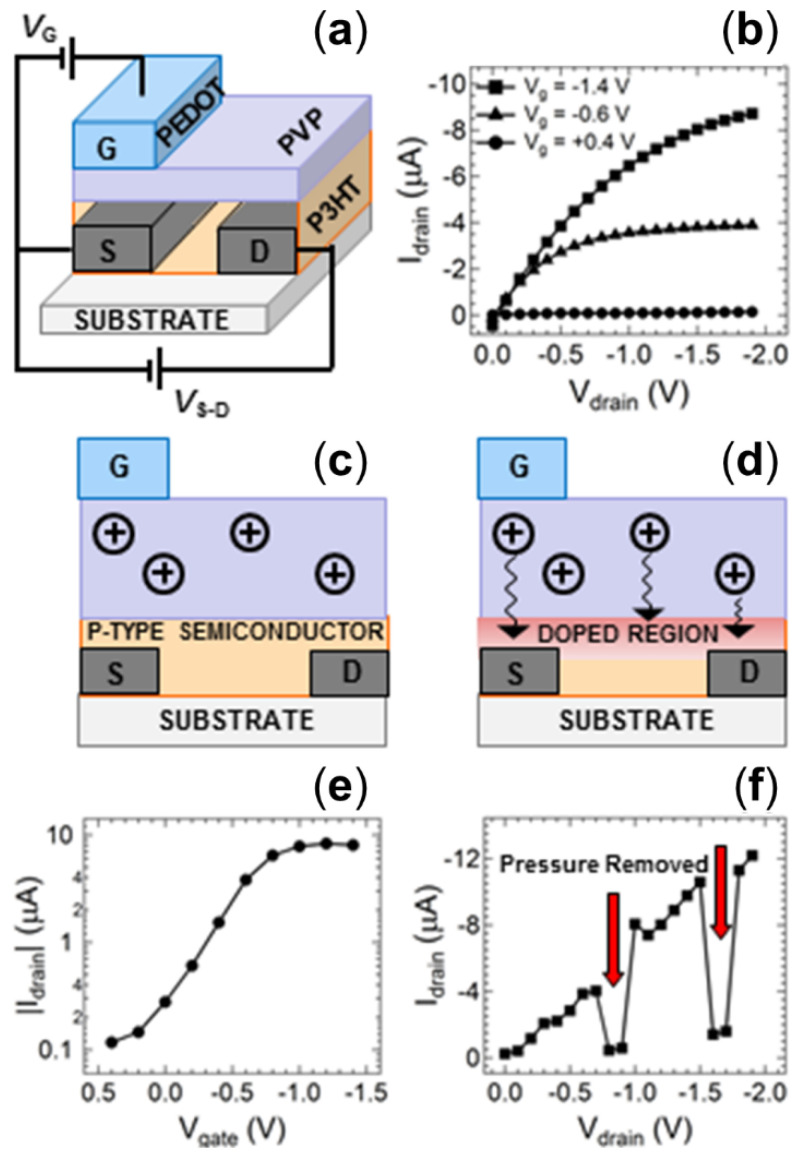
(**a**) Schematic diagram of the organic thin film transistor (OTFT) structure. (**b**) Output characteristics of the OTFT devices at various applied gate voltages. The operating mechanism of the transistor showing the device (**c**) without any applied bias, and (**d**) the movement of protons to dope the p-type P3HT channel upon application of a gate voltage. (**e**) Transfer curve of the standard OTFT at a drain voltage of −1.5 V. (**f**) Current–voltage sweep of the OTFT with an input source voltage of −1.5 V indicating the device response to a change in applied pressure at the QTC sensor. Red arrows show periods where manual pressure applied to the sensor was relaxed.

To convert the conductivity response of the QTC pressure sensor into an electronic output signal for multiplexed energetic event initiation timing, the sensor was coupled to an organic thin-film transistor (OTFT) fabricated entirely from solution-based polymers. P3HT was employed as the p-type semiconducting channel, PVP as the dielectric layer and PEDOT:PSS as the gate electrode (Figure 3a). This polymeric OTFT can therefore be readily fabricated on low-cost flexible substrates using high speed printing techniques. Such potential for low-cost fabrication makes this organic electronic device ideally suited to single use disposable applications. The output characteristic for a control device prepared using standard spin coating techniques is shown in Figure 3b. The characteristics display clearly defined linear and saturation regimes with applied gate voltages below −1.4 V, indicating successful low-voltage operation of the transistor structure. The low operating voltage of this transistor structure is a significant feature for the application of energetic event detonation, as it allows these devices to meet the low power requirements generally observed in environments with sensitive chemicals or electromagnetic interference [54]. The low-voltage operation is enabled by an electrochemical operating mechanism rather than the typical field effect observed in inorganic thin film transistors. In short, the gate voltage applied to the PEDOT electrode drives the movement of positive ions through the PVP dielectric, with these ions subsequently doping the p-type P3HT semiconductor to alter the conductivity of the semiconducting channel (Figure 3c,d) [55,56]. Such ionic movement requires much lower driving voltages than typical electrostatic field-induced doping, confirmed by the extraordinarily low −0.3 V turn-on voltage of the device in the transfer characteristic of the transistor (Figure 3e). Whilst this low turn-on voltage is a significant advantage for environments where electromagnetic signals interfere with operations, [29] there is also an associated risk of electromagnetic interference with such small signals. However, the OTFT switches the voltage signal into an inherently amplified current output signal (on/off ratio of ~10^2^ from the transfer characteristic). The electromagnetic interference risks are therefore negated since they are much more susceptible for voltage outputs than induced current outputs.

We have previously demonstrated that an OTFT fabricated using the same conditions as this control device can be used as a pressure detector by utilizing an external voltage divider circuit with a quantum tunnelling composite (QTC) material acting as a switching element [23]. In order to convert this previous device into a single integrated printed detector unit, the QTC material must be solution processed, unlike the solid films obtained commercially. For this purpose, a new QTC sensor was developed that was prepared as a solution to ensure the ability to cast and print the material as desired. The concept of the commercial material with an elastomer matrix impregnated by conducting particles was maintained, with graphite particles loaded into a silicone elastomer matrix at a solids content of 35%. Drop cast and spin-coated films prepared using this QTC solution exhibited a resistance of 5 Ω under an applied pressure of 250 kPa, consistent with the value obtained from the commercial QTC samples (Figure 2b). Figure 3f shows the output characteristic of the control OTFT with a supply voltage of −1.6 V applied to the same voltage divider circuit reported previously and manual pressure applied intermittently to a solution processed QTC sensor. The output characteristic shows an increasing negative current response from the OTFT when the QTC sensor is manually compressed and the source-drain voltage (*V*_d_) is systematically increased. This response is consistent with the output characteristic of the control device shown in Figure 3b. When the pressure on the sensor is relaxed, indicated by the red arrows in Figure 3f, the OTFT current response is reduced by more than 90%. This result verifies that removal of a pressure signal from the customized solution processed QTC sensor deactivates the OTFT current response in the same manner as we have previously reported for commercial QTC materials. The data in Figure 3f are clear evidence that both the OTFT and the QTC materials can function effectively using fully solution processed materials. This result demonstrates the ability to introduce printing fabrication techniques to integrate both the sensor and detector components into a single low-cost device.

**Figure 4:**
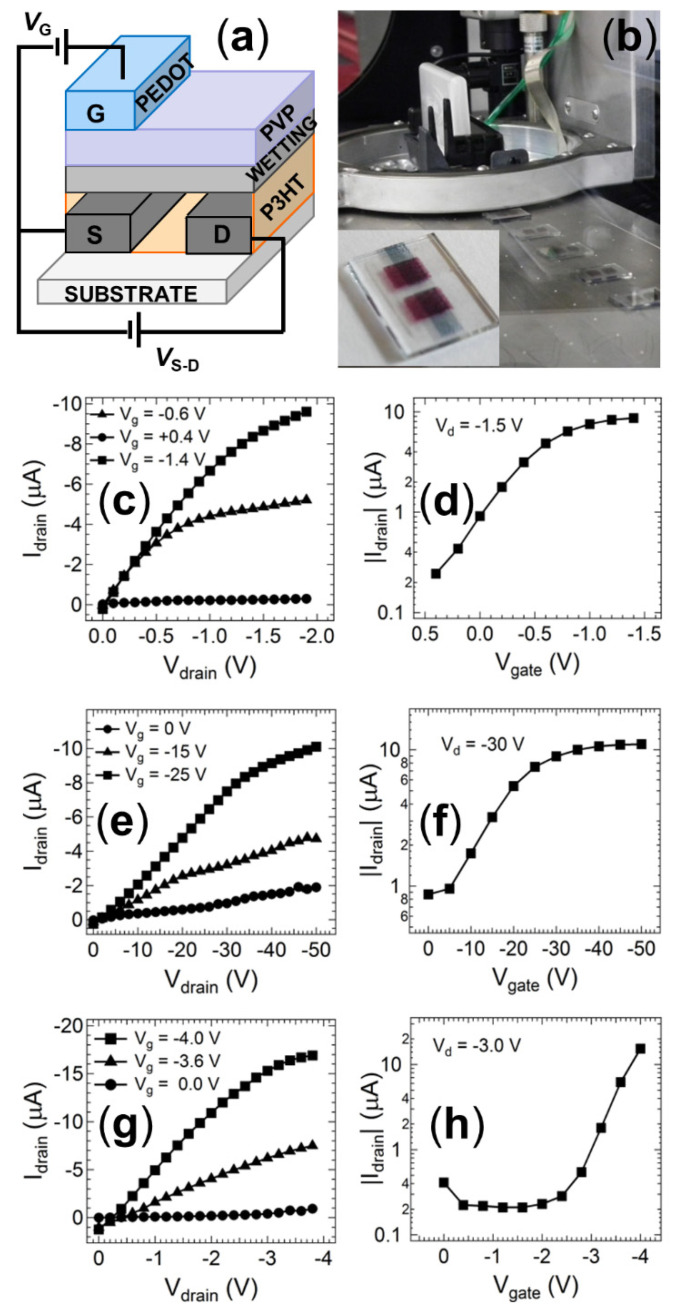
(**a**) Schematic diagram of the modified organic thin film transistor (OTFT) structure employed for inkjet-printed samples. (**b**) Photographs of the inkjet fabrication of multiple samples simultaneously. The inset shows a magnified view of a fully printed OTFT. (**c**) Output characteristic and (**d**) transfer characteristics of the OTFT structure shown in (**a**) fabricated using spin-coated films with a ZnO wetting layer. (**e**) Output characteristic and (**f**) transfer characteristics of the OTFT structure shown in (**a**) fabricated using inkjet-printed films with a ZnO wetting layer. (**g**) Output characteristic and (**h**) transfer characteristics of the OTFT structure shown in (**a**) fabricated using inkjet-printed films with a PVP wetting layer printing from 2-propanol.

**Figure 5:**
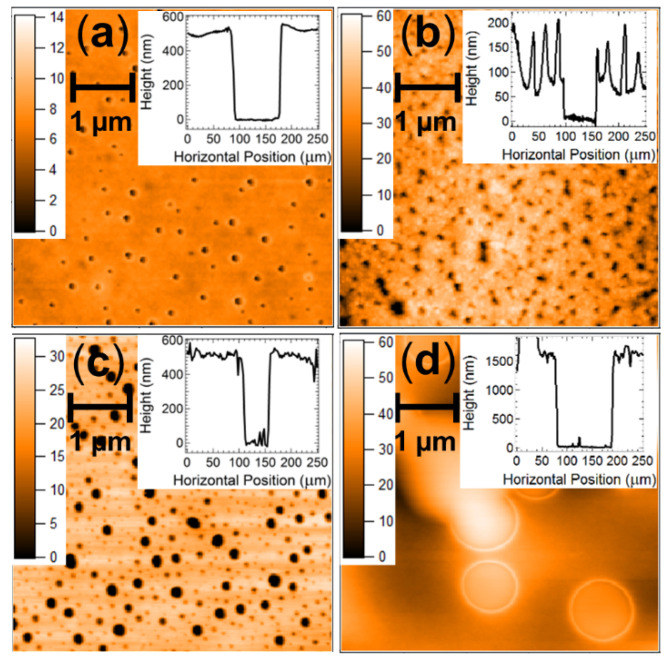
AFM data measured across a size scale of 5 × 5 µm for (**a**) spin-coated PVP films, (**b**) inkjet-printed ZnO films, (**c**) an inkjet-printed PVP wetting layer deposited from 2-propanol, and (**d**) an inkjet-printed PVP dielectric films deposited onto the inkjet-printed PVP wetting layer. The inset show profilometry data for the surface of each film with a step indicating the total film thickness.

A fully printed prototype device was fabricated using inkjet printing on a Dimatix 2800 drop-on-demand set-up (Figure 4a). Whilst the spin-coated devices with this structure exhibited good electrical responses (Figure 4b), the initial ink-jet printed devices produced non-functional OTFT sensors that exhibited short-circuiting characteristics. Analysis of the PVP dielectric layer using atomic force microscopy (AFM) revealed that the transition from spin-coating to the slower inkjet printing fabrication method creates a PVP layer that is of similar thickness but far more porous when deposited using inkjet printing (Figure 5a,c). This increased porosity arises from the mismatched surface energies created at the P3HT/PVP interface when attempting to deposit a non-polar material from polar solvent 2-propanol onto the polymer layer deposited from non-polar organic solvents. The surface tension of P3HT films has been reported to be in the range 30–36 mNm^−1^ from contact angle measurements, [57,58,59] whilst that of PVP in polar solvents is 21–24 mNm^−1^, [60] supporting the incompatibility of these two layers in slow-drying deposition techniques such as inkjet printing. The orthogonal solvents are required to prevent the second PVP layer from re-dissolving the first P3HT layer during fabrication. Spin-coating can deposit the layers from orthogonal solvents due to the fast fabrication speed that causes the solvent to rapidly evaporate and quenches the entire PVP layer into a relatively uniform film despite deposition from a non-ideal solvent. However, the slower drop-by-drop formation of the PVP layer in the inkjet printer places stronger constraints on the film formation, allowing the PVP to form into porous non-uniform films as the molecules rearrange on the much slower fabrication time scales. These porous films then facilitate shorting behaviour when the PEDOT:PSS gate electrode is applied to the porous film.

To circumvent this issue, an intermediate wetting layer between the P3HT semiconductor and PVP dielectric was deposited from materials that more readily disperse in the orthogonal solvent. ZnO was initially selected for this layer due to its reports in the literature that it can act as a strong dielectric for organic transistors based on p-type semiconductors. Following deposition of the ZnO, the PVP dielectric layer was deposited using hexanol, which is a much more effective solvent for the non-polar PVP. This new ZnO interlayer does, however, have the potential to interfere with the dipole at the semiconductor interface and thus influence the ion transport that modulates the transistor current. To examine this, a reference device was prepared with spin coating fabrication using the same layer geometry shown in Figure 4a. An I-V scan of this reference device shows that the transistor response is not affected by the thin ZnO interlayer, with a standard saturating current response (Figure 4c) and transfer characteristic (Figure 4d) observed. The magnitude of this current is very close to that observed for reference devices without the ZnO interlayer, confirming that the OTFT response is not significantly influenced by this additional layer required in the printed structure. An I-V scan of the fully inkjet-printed OTFT indicates that a standard saturating response is successfully achieved from the device (Figure 4e). Thus, the detecting and signalling elements can be fabricated using standard printing techniques. However, we note that the printed OTFT saturation response requires a larger applied gate and source-drain potential than that required for the reference device (Figure 4f). This issue is due to the imperfect nature of the printed dielectric layer, which exhibits a high surface roughness (Figure 5b) and is likely too thick to allow efficient modulation of the ionic charge at low bias voltages (other devices employing ZnO as a charge or ion transport layer have found optimum thickness values of ~30 nm). To address this issue, we amended the wetting layer to be made from PVP deposited from 2-propanol, prior to depositing a more uniform PVP layer from hexanol. An I-V scan of this new device architecture revealed a good transistor response (Figure 4h), with the magnitude of the currents comparable to the benchmark reference spin-coated devices without the interlayer, and the fully inkjet-printed transistor structure operated at gate voltages and source-drain bias voltages below 4V (Figure 4h). Measurement of the dual PVP interlayer-dielectric film with AFM and profilometry revealed a much thicker film than the spin-coated devices (1.5 µm vs. 500 nm). However, the film was now uniform and did not show any evidence of the porosity observed in the PVP films deposited solely from 2-propanol (Figure 5d). This result confirms that the thickness of the PVP layer is not critical for achieving efficient transistor responses at low bias but rather the interfacial fluid mechanics and film nanostructure must be optimised to achieve successful fully printed OTFT devices.

### 3.3. Integrating the QTC Sensor into the OTFT

**Figure 6:**
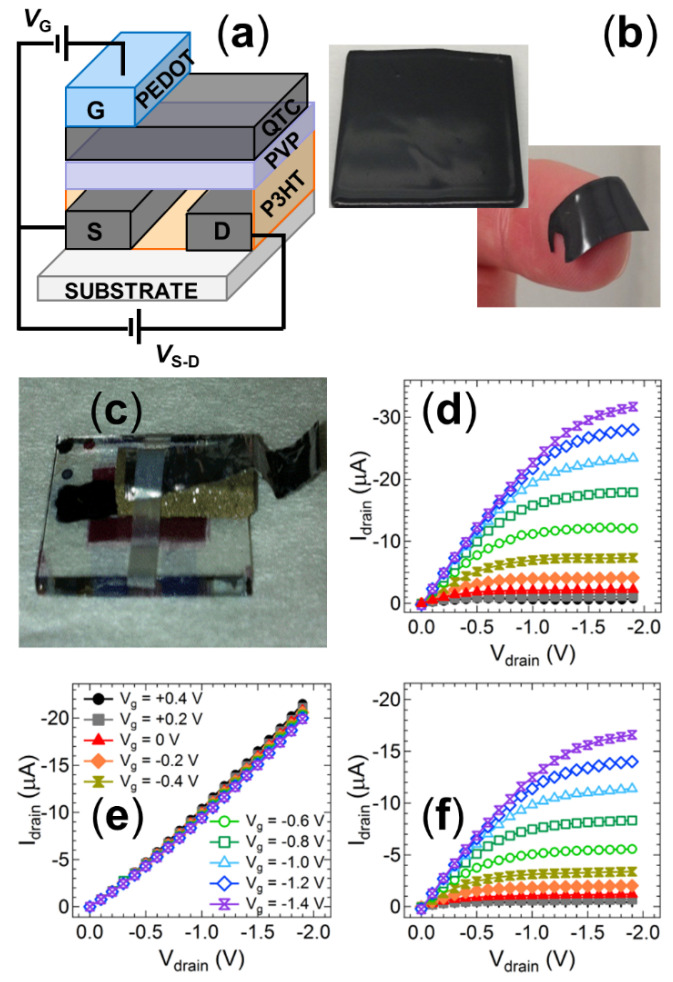
(**a**) Schematic diagram of the OTFT structure incorporating a laminated QTC sensing unit between the dielectric and gate electrode. (**b**) Photographs of the solution processed printable QTC layer. (**c**) Photograph of the fully solution processed printable pressure sensor device. Current–voltage sweeps from an OTFT device with (**d**) no QTC sensor layer incorporated, (**e**) a laminated QTC between dielectric and gate electrode with no transmission fuse output, and (**f**) a laminated QTC between dielectric and gate electrode after transmission fuse output activates the sensor.

After independently establishing the feasibility of the printed OTFT detector and the solution processed QTC sensor, efforts were directed towards incorporating the QTC sensing unit onto the transistor structure to produce a fully printable and integrated sensing and signalling system. The structure of this integrated device and photos of the solution processed QTC films cast into a thin flexible layer that can be laminated or printed onto the dielectric material and the fully assembled device are shown in Figure 6a–c, respectively. The output characteristics of a control OTFT device fabricated using spin cast layers and prepared with and without a spin cast QTC barrier layer are shown in Figure 6c,d. Spin cast layers were employed for this integrated structure as the solution processed QTC cannot be fabricated with inkjet printing due to viscosity limitations with the current formulation (η > 2000 mPa·s). The viscosity and surface tension of inks are known to be of critical importance for achieving good inkjet printability. For the Fuji Dimatix printer employed in these studies, the range for optimal jetting performance is 28–33 nMm^−1^ for surface tension and 10–12 mPa·s for viscosity. Thus, the inks employed to fabricate the OTFT fall within this range and can be printed, whilst the solution cast QTC has a viscosity more than two orders of magnitude higher than the range required for optimal inkjet printability and cannot be fabricated with this technique. The introduction of the QTC layer into the device results in a loss of modulation of the OTFT. This result is consistent with the high resistivity of the QTC layer in an uncompressed state, resulting in the applied gate voltage being unable to influence the dielectric layer as the voltage drop across the resistive QTC layer is too high. In this condition, the device behaves as an Ohmic resistor, with a linear relationship between the drain current and voltage independent of the gate voltage. The conductivity of the P3HT is significantly increased in the presence of a PVP layer as previously reported, [58] leading to a measured resistance of 90 kΩ. When the transmission fuse is initiated to supply a pressure output to the QTC, the sensing unit is compressed into its conductive state, allowing the gate voltage to be applied to the dielectric and the modulation of the current returns (Figure 6e).

**Figure 7:**
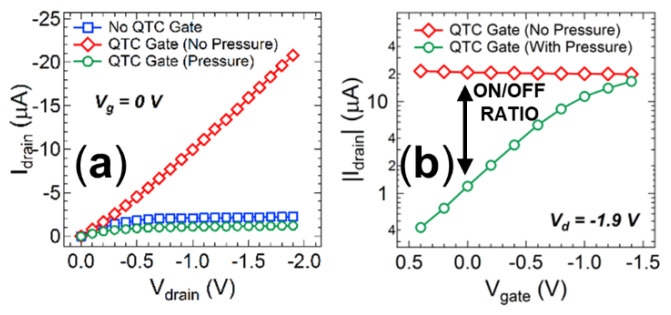
(**a**) A comparison of the current–voltage sweeps with no applied gate voltages for an OTFT device with no QTC sensor layer incorporated, a laminated QTC between dielectric and gate electrode with no transmission fuse output, and a laminated QTC between dielectric and gate electrode after transmission fuse output activates the sensor. (**b**) A comparison of the transistor transfer curves in the on (no pressure) and off (with pressure) states at a drain voltage of −1.9 V.

Since the OTFT behaviour is completely resistive without pressure applied to the QTC, the current response will continue to increase linearly with increasing source-drain voltage. Consequently, the OTFT response without pressure will always be larger than the response observed when pressure is applied to the device and the current saturates, independent of the gate voltage utilized. The fully integrated printable system is therefore more suited to function as a normally on switch, with an on-off ratio that is continually increasing as the source-drain voltage is increased (Figure 7a). This feature can provide a significant advantage, as it removes the requirement for biasing the gate electrode, and therefore eliminates the potential influence of electromagnetic interference with this power supply. A simple conductive connection printed between the gate and source electrodes will ensure this 0 V gate voltage in practical operation. In this configuration, the OTFT produces a drain current of 20.8 µA without pressure, dropping to a drain current of 1.1 µA when pressure is detected from the transmission fuse (Figure 7b). The integrated printable sensing and signalling system can therefore function as an efficient and cheap initiation system that detects a pressure input and converts it to an electrical switching signal with the only external requirement being source-drain biasing from a low voltage battery.

## 4. Conclusions

In this paper, we have described the development of an integrated and printable organic electronic sensing and signalling system to convert the pressure output of transmission fuse tubing into an electronic trigger signal for the precise detonation of multiple energetic events. The detector was composed of two critical units; a quantum tunnelling composite material to act as a sensor for the pressure pulse, and an organic thin film transistor to convert the mechanical pressure wave into an electronic current signal with inherent amplification and ease of integration to further circuitry. The quantum tunnelling composite material displayed a rapid switching from a non-conductive to a conductive state upon application of pressure levels 2 orders of magnitude lower than those available from the transmission fuse output. The material was subsequently utilized to moderate the gate voltage of an all-polymer OTFT. Pressure output from the transmission fuse, coupled to the sensor through a simple piston arrangement, was able to increase the current output of the device by more than an order of magnitude to provide a normally off switch. It was subsequently demonstrated that the all-polymer OTFT could be fabricated using inkjet printing. This approach provides a viable route towards fabrication on low cost and flexible substrates. It was then shown that the QTC sensor could be integrated into the transistor by casting from a solution to provide a single device structure that can be fabricated entirely from printing techniques. Pressure output from the transmission fuse decreased the current output of this integrated device by more than an order of magnitude without the need for any gate voltage power supply, providing a normally on switch. The fabrication of a fully printable low-cost pressure detector opens up exciting opportunities for the development of cheap and simple assemblies for a range of different applications.

## Data Availability

Data is available upon reasonable request from the corresponding author.

## References

[1] Briseno A.L., Mannsfeld S.C.B., Jenekhe S.A., Bao Z., Xia Y. (2008). Introducing Organic Nanowire Transistors. Mater. Today.

[2] Lee M.Y., Lee H.R., Park C.H., Han S.G., Oh J.H. (2018). Organic Transistor-Based Chemical Sensors for Wearable Bioelectronics. Acc. Chem. Res..

[3] Griffith M.J., Holmes N.P., Elkington D.C., Cottam S., Stamenkovic J., Kilcoyne A.L.D., Andersen T.R. (2020). Manipulating nanoscale structure to control functionality in printed organic photovoltaic, transistor and bioelectronic devices. Nanotechnology.

[4] Ameri M., Al-Mudhaffer M.F., Almyahi F., Fardell G.C., Marks M., Al-Ahmad A., Fahy A., Andersen T., Elkington D.C., Feron K. (2019). Role of Stabilizing Surfactants on Capacitance, Charge, and Ion Transport in Organic Nanoparticle-Based Electronic Devices. ACS Appl. Mater. Interfaces.

[5] Kergoat L., Piro B., Berggren M., Horowitz G., Pham M.-C. (2012). Advances in Organic Transistor Based Biosensors: From Organic Electrochemical Transistors to Electrolyte-Gated Organic Field-Effect Transistors. Anal. Bioanal. Chem..

[6] Marks M., Holmes N.P., Sharma A., Pan X., Chowdhury R., Barr M.G., Fenn C., Griffith M.J., Feron K., Kilcoyne A.L.D. (2019). Building intermixed donor-acceptor architectures for water-processable organic photovoltaics. Phys. Chem. Chem. Phys..

[7] Wang L., Fine D., Sharma D., Torsi L., Dodabalapur A. (2006). Nanoscale organic and polymeric field-effect transistors as chemical sensors. Anal. Bioanal. Chem..

[8] Griffith M.J., Sunahara K., Furube A., Mozer A.J., Officer D.L., Wagner P., Wallace G.G., Mori S. (2013). Cation Exchange at Semiconducting Oxide Surfaces: Origin of Light-Induced Performance Increases in Porphyrin Dye-Sensitized Solar Cells. J. Phys. Chem. C.

[9] Hösel M., Krebs F.C. (2012). Large-scale roll-to-roll photonic sintering of flexo printed silver nanoparticle electrodes. J. Mater. Chem..

[10] Griffith M.J., Cooling N.A., Vaughan B., Elkington D.C., Hart A.S., Lyons A.G., Quereshi S., Belcher W.J., Dastoor P.C. (2016). Combining Printing, Coating, and Vacuum Deposition on the Roll-to-Roll Scale: A Hybrid Organic Photovoltaics Fabrication. IEEE J. Sel. Top. Quantum Electron..

[11] Griffith M.J., Cooling N.A., Vaughan B., O’Donnell K.M., Al-Mudhaffer M.F., Al-Ahmad A., Noori M., Almyahi F., Belcher W.J., Dastoor P.C. (2015). Roll-to-Roll Sputter Coating of Aluminum Cathodes for Large-Scale Fabrication of Organic Photovoltaic Devices. Energy Technol..

[12] Andersen T.R., Almyahi F., Cooling N.A., Elkington D., Wiggins L., Fahy A., Feron K., Vaughan B., Griffith M.J., Mozer A.J. (2016). Comparison of inorganic electron transport layers in fully roll-to-roll coated/printed organic photovoltaics in normal geometry. J. Mater. Chem. A.

[13] Baran D., Ashraf R.S., Hanifi D.A., Abdelsamie M., Gasparini N., Rohr J.A., Holliday S., Wadsworth A., Lockett S., Neophytou M. (2017). Reducing the efficiency-stability-cost gap of organic photovoltaics with highly efficient and stable small molecule acceptor ternary solar cells. Nat. Mater..

[14] Holmes N.P., Vaughan B., Williams E.L., Kroon R., Anderrson M.R., Kilcoyne A.L.D., Sonar P., Zhou X., Dastoor P.C., Belcher W.J. (2017). Diketopyrrolopyrrole-based polymer:fullerene nanoparticle films with thermally stable morphology for organic photovoltaic applications. MRS Commun..

[15] Yin W., Dadmun M. (2011). A new model for the morphology of P3HT/PCBM organic photovoltaics from small-angle neutron scattering: Rivers and streams. ACS Nano..

[16] Zhou L., He X., Lau T.K., Qiu B., Wang T., Lu X., Luszczynska B., Ulanski J., Xu S., Chen G. (2018). Nonhalogenated Solvent-Processed All-Polymer Solar Cells over 7.4% Efficiency from Quinoxaline-Based Polymers. ACS Appl. Mater. Interfaces.

[17] Cho J.H., Lee J., Xia Y., Kim B., He Y., Renn M.J., Lodge T.P., Frisbie C.D. (2008). Printable ion-gel gate dielectrics for low-voltage polymer thin-film transistors on plastic. Nat. Mater..

[18] Elkington D., Wasson M., Belcher W., Dastoor P.C., Zhou X. (2015). Printable organic thin film transistors for glucose detection incorporating inkjet-printing of the enzyme recognition element. Appl. Phys. Lett..

[19] Maasoumi F., Ullah M., Shaw P.E., Li J., Burn P.L., Meredith P., Namdas E.B. (2015). Charge transport and recombination in heterostructure organic light emitting transistors. Org. Electron..

[20] Someya T., Sekitani T., Iba S., Kato Y., Kawaguchi H., Sakurai T. (2004). A large-area, flexible pressure sensor matrix with organic field-effect transistors for artificial skin applications. Proc. Natl. Acad. Sci. USA.

[21] Mabrook M.F., Pearson C., Petty M.C. (2006). Inkjet-Printed Polymer Films for the Detection of Organic Vapors. IEEE Sens. J..

[22] Thompson B., Yoon H.-S. (2013). Aerosol-Printed Strain Sensor Using PEDOT:PSS. IEEE Sens. J..

[23] Griffith M.J., Cooling N.A., Elkington D.C., Muller E., Belcher W.J., Dastoor P.C. (2014). Printable Sensors for Explosive Detonation. Appl. Phys. Lett..

[24] Sekitani T., Someya T. (2010). Stretchable, Large-area Organic Electronics. Adv. Mater..

[25] Someya T., Kato Y., Iba S., Noguchi Y., Sekitani T., Kawaguchi H., Sakurai T. (2005). Integration of organic FETs with organic photodiodes for a large area, flexible, and lightweight sheet image scanners. IEEE Trans. Electron. Dev..

[26] Sekitani T., Noguchi Y., Zschieschang U., Klauk H., Someya T. (2008). Organic transistors manufactured using inkjet technology with subfemtoliter accuracy. Proc. Natl. Acad. Sci. USA.

[27] Babu A.S., Mishra K.K., Kshirsagar P.D., Shekhar H., Rasane V.S. (2013). Programmable Electronic Delay Device for Detonator. Defence Sci. J..

[28] Kichouliya R., Devender R., Ramasarma V.V., Reddy D.S., Borkar V.G. Hazards of electromagnetic radiation to ordnance (HERO) assessment of electro-explosive devices and validation of extrapolation method for estimation of the safety margin at HERO electromagnetic environments. Proceedings of the 2011 IEEE International Symposium on Electromagnetic Compatibility.

[29] Pantoja J.J., Peña N.M., Rachidi F., Vega F., Roman F. (2013). Susceptibility of Electro-explosive Devices to Microwave Interference. Defence Sci. J..

[30] Mishra P.K., Miodrag B., Mustapha C.E., Yagoub S.R.F. (2012). RFID technology for tracking and tracing explosives and detonators in mining services applications. J. Appl. Geophys..

[31] Smit F.C., Pistorius C.W.I. (1998). Implications of the Dominant Design in Electronic Initiation Systems in the South African Mining Industry. Technol. Forecast. Soc..

[32] Zhu P., Shen R., Fiadosenka N.N., Ye Y., Hu Y. (2011). Dielectric structure pyrotechnic initiator realized by integrating Ti/CuO-based reactive multilayer films. J. Appl. Phys..

[33] Freiwald D.A. (1972). Approximate Blast Wave Theory and Experimental Data for Shock Trajectories in Linear Explosive-Driven Shock Tubes. J. Appl. Phys..

[34] Westbrook C.K. (1979). An Analytical Study of the Shock Tube Ignition of Mixtures of Methane and Ethane. Combust. Sci. Technol..

[35] Samuelraj I.O., Jagadeesh G., Kontis K. (2013). Micro-blast waves using detonation transmission tubing. Shock Waves.

[36] Mabeck J.T., Malliaras G.G. (2006). Chemical and biological sensors based on organic thin-film transistors. Anal. Bioanal. Chem..

[37] Samuel I.D.W. (2000). Polymer Electronics. Phil. Trans. Royal Soc. A.

[38] Cho B., Kim T.-W., Song S., Ji Y., Jo M., Hwang H., Jung G.-Y., Lee T. (2010). Rewritable Switching of One Diode-One Resistor Nonvoltaile Organic Memory Devices. Adv. Mater..

[39] DeFranco J.A., Schmidt B.S., Lipson M., Malliaras G.G. (2006). Photolithographic Patterning of Organic Electronic Materials. Org. Electron..

[40] Abdolmaleki H., Kidmose P., Agarwala S. (2021). Droplet-based techniques for printing of functional inks for flexible physical sensors. Adv. Mater..

[41] Kamyshny A., Magdassi S. (2014). Conductive nanomaterials for printed electronics. Small.

[42] Gans B.J., Duineveld P.C., Schubert U.S. (2004). Inkjet printing of polymers: State of the art and future developments. Adv. Mater..

[43] Abdolmaleki H., Agarwala S. (2020). PVDF-BaTiO_3_ nanocomposite inkjet inks with enhanced phase crystallinity for printed electronics. Polymers.

[44] Yu G., Cao Y., Wang J., McElvain J., Heeger A.J. (1999). High sensitivity polymer photosensors for image sensing applications. Synth. Met..

[45] Labram J., Wöbkenberg P., Bradley D., Anthopoulos T. (2010). Low-voltage ambipolar phototransistors based on a pentacene/PC61BM heterostructure and a self-assembled nano-dielectric. Org. Electron..

[46] Manunza I., Sulis A., Bonfiglio A. (2006). Pressure sensing by flexible, organic, field effect transistors. Appl. Phys. Lett..

[47] Manunza I., Bonfiglio A. (2007). Pressure sensing using a completely flexible organic transistor. Biosens. Bioelectron..

[48] Jing X., Zhao W., Lan L. (2000). The effect of particle size on electric conducting percolation threshold in polymer/conducting particle composites. J. Mater. Sci. Lett..

[49] Ounaies Z., Park C., Wise K.E., Siochi E.J., Harrison J.S. (2003). Electrical properties of single wall carbon nanotube reinforced polyimide composites. Compos. Sci. Technol..

[50] Hwang M., Lee H.S., Jang Y., Cho J.H., Lee S., Kim D.H., Cho K. (2009). Effect of curing conditions of a poly(4-vinylphenol) gate dielectric on the performance of a pentacene-based thin film transistor. Macromol. Res..

[51] Forde L.C., Proud W.G., Walley S.M., Church P.D., Cullis I.G. (2010). Ballistic impact studies of a borosilicate glass. Int. J. Impact Eng..

[52] Sutton D., Noble A.H., Lynch P.M. Studies of Explosive Shock Tubes. Proceedings of the 14th International Pyrotechnic Seminar.

[53] Tsang D.K.L. (2006). A numerical study of a detonation wave in detonation transmission tubing. Math. Comp. Model..

[54] Eckhoff R.K. (2002). Minimum ignition energy (MIE)—A basic ignition sensitivity parameter in design of intrinsically safe electrical apparatus for explosive dust clouds. J. Loss Prevent. Proc. Indust..

[55] Elkington D., Darwis D., Zhou X., Belcher W., Dastoor P.C. (2012). The fabrication and characterization of poly(4-vinylpyridine)-based thin film transistors exhibiting enhanced ion modulation. Org. Electron..

[56] Bäcklund T.G., Österbacka R., Stubb H., Bobacka J., Ivaska A. (2005). Operating principle of polymer insulator organic thin-film transistors exposed to moisture. J. Appl. Phys..

[57] Jaczewska J., Raptis I., Budkowski A., Goustouridis D., Raczkowska J., Sanopoulou M., Pamula E., Bernasik A., Rysz J. (2007). Swelling of poly(3-alkylthiophene) films exposed to solvent vapors and humidity: Evaluation of solubility parameters. Synth. Met..

[58] Destri G.L., Keller T.F., Catellani M., Punzo F., Jandt K.D., Marletta G. (2011). Crystalline monolayer ordering at substrate/polymer interfaces in poly(3-hexylthiophene) ultrathin films. Macromol. Chem. Phys..

[59] Kim H., Yoon B., Sung J., Choi D.G., Park C. (2008). Micropatterning of thin P3HT films via plasma enhanced polymer transfer printing. J. Mater. Chem..

[60] Taghizadeh M., Amiri S.S. (2017). Experimental measurements and modelling of the solvent activity and surface tension of binary mixtures of poly(vinyl pyrrolidone) in water and ethanol. J. Serb. Chem. Soc..

